# Intranasal Resveratrol Plus Carboxymethyl-β-Glucan: A Promising Option in Clinical Practice

**DOI:** 10.3390/biom16020285

**Published:** 2026-02-11

**Authors:** Giorgio Ciprandi, Maria Angela Tosca

**Affiliations:** 1 Allergy Clinic, Casa di Cura Villa Montallegro, 16100 Genoa, Italy; 2 Allergy Center, IRCCS Istituto Giannina Gaslini, 16100 Genoa, Italy

**Keywords:** trans-resveratrol, carboxymethyl-β-glucan, respiratory infections, allergic rhinitis, nasal surgery

## Abstract

Resveratrol is a polyphenol with numerous beneficial properties, acting as a phytoalexin. Plants produce resveratrol in response to various types of stress, such as infections and external damage. Resveratrol is a natural medicine thanks to its antimicrobial, antioxidant, anti-inflammatory, immunomodulatory, and antiallergic properties. However, resveratrol has poor oral bioavailability. To overcome this drawback, a topical nasal formulation has been developed, characterized by the fixed combination of carboxymethyl-β-glucan (CMBG) with trans-resveratrol. This innovative molecule has been the subject of in-depth preclinical studies to demonstrate its stability and solubility. Subsequently, several controlled clinical studies have shown that this formulation is effective and safe in patients with respiratory infections, allergic rhinitis, and nasal surgery. Therefore, taken together, these findings suggest that intranasal resveratrol–CMBG might be a promising option for the management of upper airway diseases.

## 1. Introduction

Resveratrol (3,5,4′-trihydroxy-trans-stilbene) is a non-flavonoid phenol [1]. The phenol compounds are aromatic compounds derived from benzene with a hydroxyl group (-OH) directly attached to the benzene ring. On the other hand, flavonoids are water-soluble polyphenolic compounds that are secondary plant metabolites, usually present as glycosides. Resveratrol is also a phytoalexin produced naturally by numerous plants in response to attacks by pathogens such as bacteria or fungi [1]. In fact, phytoalexins are antimicrobial compounds produced in response to pathogen interactions; they are consistently absent in healthy, disease-free plants.

Resveratrol was first identified in *Veratrum grandiflorum* (i.e., white hellebore) in the 1940s. Furthermore, resveratrol has been isolated from several plants, including grapes, peanuts (mainly in the peel), blueberries, and raspberries. However, the primary source of resveratrol is *Polygonum cuspidatum*, as its concentration is about 296–377 mg/g [2]. *Polygonum cuspidatum* is a plant native to East Asia and is currently widespread in North America and Europe [3]. It is a perennial herbaceous plant belonging to the *Polygonaceae* family. The plant has vigorous, hairless stems that can reach 3–4 metres in height. *Polygonum cuspidatum* produces long, sturdy underground stems (rhizomes) that can grow deep into the ground. The leaves are alternate, oval, 7–14 cm long and 5–12 cm wide, and are truncated at the base with an inner margin. The flowers are small, creamy white, and are produced in late summer and early autumn in erect racemes (clusters) 6–15 cm long. *Polygonum cuspidatum* is also called “false bamboo”, as it is a member of the Polygonaceae family. In the dry extract derived from the root of the plant, the main bioactive constituent is resveratrol [4].

Given the “defensive” nature of resveratrol, which is produced in response to harmful stimuli, it is easy to see how this molecule can perform numerous healing functions for the plant itself. Namely, resveratrol exerts antimicrobial (mainly antiviral), anti-inflammatory, antioxidant, and immunomodulant effects [5]. As a result, compounds containing resveratrol have been proposed for the treatment of several diseases, including cancer, metabolic disorders, cardiovascular diseases, neurodegenerative conditions, and inflammatory diseases [6,7]. However, the main drawback of oral resveratrol is the low bioavailability, as demonstrated by pharmacokinetic studies [8,9]. As a result, high doses are used in clinical practice, but adverse reactions may occur frequently. Therefore, trans-resveratrol is used instead of resveratrol because it is the most abundant form in nature, is more stable, is significantly more bioactive (up to six times more potent), and is better absorbed and utilized by the body [10]. However, free trans-resveratrol may not be very effective when administered topically because it is poorly absorbed and rapidly cleared [11]. Consequently, new formulations based on adequate carriers have been developed [12]. In this regard, an innovative formulation based on the association of trans-resveratrol with carboxymethyl-β-glucan has been studied to improve stability in aqueous solutions [13]. The β-glucans constitute a class of natural polysaccharides characterized by two main activities: delivery capability and immunomodulatory effects. The term β-glucans refers to non-cellulosic polymers of β-glucose, characterized by β-glycosidic bonds and β-bound glucose molecules [14]. Beta-glucans are usually isolated from fungi, cereals, bacteria, or seaweeds. Thanks to their delivery characteristic, β-glucans serve for the nanoencapsulation of lipophilic molecules, and, in particular, for poorly bioavailable compounds, e.g., resveratrol. In addition, β-glucans have significant positive effects on immunity, to the point that they were considered biological response modifiers [15]. This immunomodulatory property also confers antioxidant and wound-healing activities [16].

**Figure 1:**
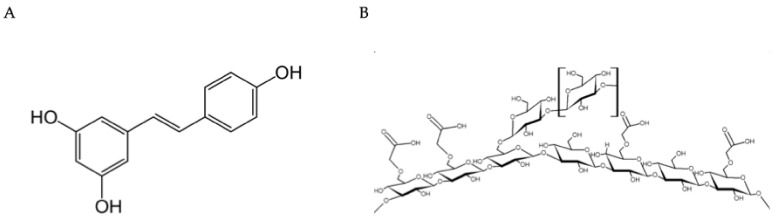
Molecular structure and formulation. (**A**) Trans-resveratrol. A non-flavonoid phenol (3,5,4′-trihydroxy-trans-stilbene) and phytoalexin produced by plants in response to pathogens. Primary source: *Polygonum cuspidatum* (296–377 mg/g concentration). (**B**) Carboxymethyl-β-Glucan. A modified polysaccharide with enhanced water solubility and stability. Provides delivery capability and immunomodulatory effects while maintaining long-term stability when combined with resveratrol.

To improve water solubility and stability, carboxymethylation of β-glucans is a widely used chemical procedure for developing topical products. Starting with natural beta-glucan, the hydroxyl groups in the polysaccharide are chemically modified with carboxymethyl groups (-CH_2_-COOH) to make the product more water-soluble, more stable, and easier to use in topical (intranasal) formulations, thereby conferring moisturizing, protective, and regenerative properties for the epithelial mucosa. Figure 1 reports the molecular structures of the two components.

Accordingly, carboxymethylated β-glucans (CMBG) exhibit high stability and water solubility due to easily ionizable carboxyl residues [17]. Namely, it should be emphasized that the incorporation of resveratrol did not alter its biological characteristics, particularly its long-term stability and solubility [13]. A further study also reinforced these aspects, considering photochemical stability and bioavailability [18]. The same group of researchers demonstrated that trans-resveratrol–CMBG inhibited rhinovirus replication and the expression of pro-inflammatory mediators (IL-6, IL-8, and RANTES) in infected human epithelial cell lines in vitro [19]. In addition, resveratrol–CMBG decreased their expression of ICAM-1, an adhesion molecule ligand of β-integrins [20]. Trans-resveratrol–CMBG was also studied to develop an aerosol formulation capable of reaching the lower airways [21]. In particular, the mass median aerodynamic diameter was lower than that of their parental molecules alone, suggesting that this association can penetrate the entire respiratory tree. Another study provided interesting evidence confirming the close cooperation between CMBG and resveratrol [22]. This study showed that the presence of resveratrol increased the CMBG structure in a water solution. Thus, this finding suggests that resveratrol may render the polymer more stable, thereby promoting long-term stability of the CMBG–resveratrol complex.

Table 1 summarizes the preclinical studies that investigated the CMBG–resveratrol formulation.

Based on these general considerations and biological evidence, the trans-resveratrol–CMBG combination has been the subject of several clinical studies involving children with respiratory infections and allergies, as highlighted by some publications. However, these papers did not thoroughly examine the specific mechanisms of action of the intranasal combination of resveratrol and CMBG, particularly, the activity of CMBG and the anti-inflammatory, antimicrobial (mainly antiviral), and antiallergic activities of resveratrol. In addition, the present narrative review updates and discusses the most recent publications on this intranasal combination and delineates the nose scenario where CMBG–resveratrol should act. The selection of articles was essential because there is a vast number of articles published on resveratrol. In fact, searching PubMed for the keyword “resveratrol” returns over 20,000 publications.

## 2. Methods

This narrative review followed three steps: conducting the search, reviewing abstracts and full-texts, and discussing the results. To this end, the PubMed database was searched as the review progressed to identify relevant studies. The final search was conducted in January 2026 and included English-language international articles, online reports, and electronic books. The inclusion criteria considered keywords such as “intranasal CMBG–resveratrol and allergic disease”, “intranasal CMBG–resveratrol and respiratory infections”, and “intranasal CMBG–resveratrol and immune system”, which were used to search for clinical studies. Exclusion criteria were the lack of reference to the intranasal combination. After the search was complete, the abstracts were reviewed to ensure that they addressed the topic of interest. All duplicates were removed, and the abstracts of the remaining articles were reviewed to ensure they met the inclusion criteria. Eligible studies were those that investigated the intranasal combination CMBG–resveratrol across at least one of the three aspects (in vitro, animal, or human). The authors selected the pertinent studies for the present review. Therefore, these studies of interest were summarized to integrate the narrative review. Given the narrative nature, the search was not systematic; however, keywords, timeframes and databases are transparently reported.

## 3. The Nose Scenario

The nose is the gateway for inhaled air and is therefore exposed to the penetration of pathogenic germs, allergens, and irritants (pollutants, tobacco smoke, and toxic substances). For this reason, the nose can easily become the site of inflammatory processes. In this regard, it is important to emphasize that every infectious process, regardless of its nature (bacterial, viral, or fungal), triggers an inflammatory reaction. This aspect is crucial because we must always keep the infection–inflammation binomial in mind. In other words, there is no infection without inflammation.

Similarly, allergic rhinitis and non-allergic cell-mediated rhinitis (e.g., non-allergic rhinitis with eosinophils—NARES) are characterized by type 2 inflammation [24]. Therefore, even in this case, allergic and non-allergic rhinitis are inflammatory diseases. Here, too, the allergy–inflammation pairing applies, and likewise, there is no allergy without inflammation.

Another critical point is that every inflammatory process, especially if acute, causes oxidative stress [25]. Oxidative stress results from the accumulation of free radicals that are not sufficiently eliminated. There are two types of free radicals: those derived from oxygen, i.e., reactive oxygen species (ROS), and those derived from nitrogen, i.e., reactive nitrogen species (RNS) [26]. An excess of free radicals amplifies and sustains the inflammatory state that produced them. In fact, free radicals damage cells involved in the inflammatory process, further exacerbating cellular injury. Cells damaged by free radicals, precisely because they are further inflamed, cause the release of other free radicals, which in turn aggravate the pre-existing inflammation. Ultimately, this creates a vicious circle that starts with infection, progresses to inflammation, which in turn causes oxidative stress, which further aggravates the inflammation [27].

This loop also occurs in the model of allergic inflammation. In fact, exposure to the allergen triggers a cascade of inflammatory events that, in turn, induces oxidative stress [28]. A classic example of this situation is the increase in nitric oxide (NO) in the respiratory tract. NO is a free radical derived from nitrogen and is also a valuable biomarker of type 2 inflammation [29].

However, the situation is even more complex, as the immunological structure of allergic diseases is characterized by type 2 polarization [30]. This immunological imbalance leads to reduced type 1 immunity, which is responsible for defending against infections. This phenomenon is why people with allergies are more prone to infections and therefore get sick more often than people without allergies [31]. Furthermore, precisely because of this immunological imbalance, people with allergies contract more serious and longer-lasting infections [32].

**Figure 2:**
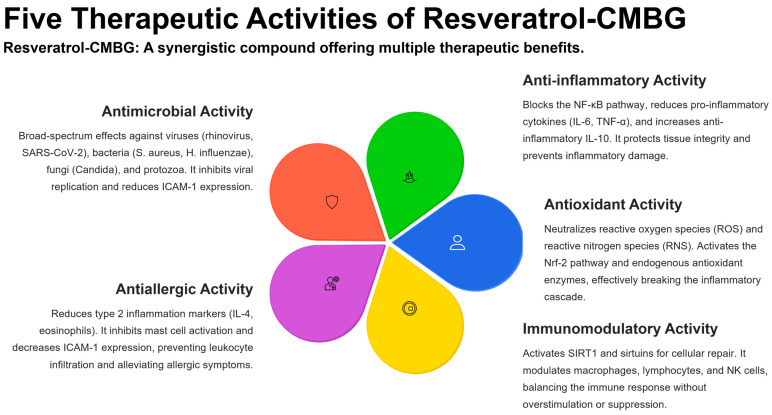
Five therapeutic activities of resveratrol–CMBG.

This situation further aggravates the vicious circle, creating a self-perpetuating vicious circle: allergy–inflammation–oxidative stress–infection–inflammation, and so on (Figure 2). However, this model represents a simplified framework and may not apply uniformly across phenotypes

Based on these considerations, derived from clinical and experimental evidence, it is important to use substances that exhibit antimicrobial, anti-inflammatory, antioxidant, and antiallergic activity. In this context, resveratrol can exert all these activities simultaneously.

## 4. Resveratrol Effects (Non-Formulation Specific)

**Figure 3:**
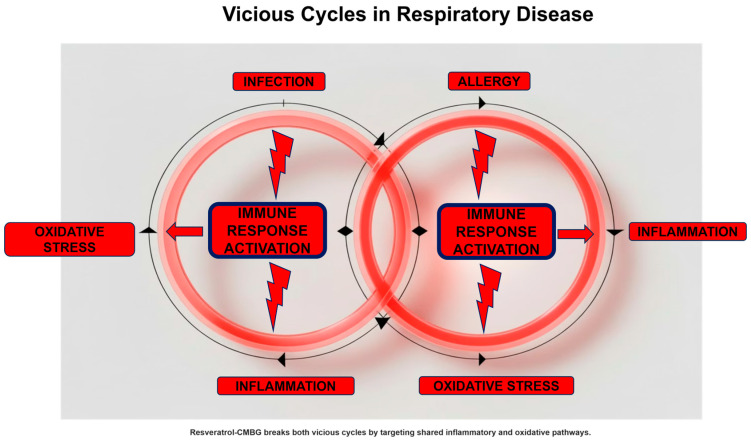
Vicious cycles in respiratory disease.

Resveratrol exerts several beneficial functions that may be advantageously used in clinical practice. These activities concern different targets, as reported below and summarized in Figure 3.

### 4.1. Antimicrobial Activity of Resveratrol

One of resveratrol’s primary functions is to defend the plant against pathogens, as it is a phytoalexin, such as a protein with cytolytic activity [33]. For this reason, its antimicrobial activity must be broad-spectrum, thus counteracting infections caused by viruses, bacteria, fungi, and protozoa.

This antibacterial activity has also been exploited in humans to treat infections, especially respiratory infections [34]. Namely, resveratrol is able to contrast Gram-positive and Gram-negative bacteria [35]. In particular, resveratrol has been used to treat infections caused by *Staphylococcus aureus*, including methicillin-resistant strains [36,37]. Interestingly, it has recently been demonstrated that resveratrol increases the antibiofilm and antibacterial activity of polymyxin B against carbapenem-resistant *Acinetobacter baumannii* [38]. In addition, resveratrol may hinder several pathogens, including *E. coli*, *Listeria* sp., *Staphylococcus aureus*, *Campylobacter* sp., and *Vibrio* sp. [39]. Resveratrol may also suppress inflammation induced by non-typeable *Haemophilus influenzae* [40]. Therefore, resveratrol, thanks to its antibacterial and antibiofilm properties, might be advantageously used as an add-on therapy to manage bacterial infections.

The antiviral activity has been extensively investigated. In particular, the COVID-19 pandemic has renewed the growing interest in this substance [41]. In particular, a clinical study demonstrated that resveratrol supplementation may reduce ACE2 expression in human adipocytes [42]. This finding might be clinically relevant, as ACE2 is a receptor for SARS-CoV-2, and its inhibition may contribute to the prevention of COVID-19 infection. Very recently, a randomized, double-blinded, placebo-controlled clinical study investigated resveratrol’s ability to modulate pro-inflammatory cytokines and biomarkers of inflammation in patients with COVID-19 infection [43]. The actively treated patients took oral resveratrol 750 mg daily for 10 days. Resveratrol supplementation significantly reduced levels of the pro-inflammatory cytokines IL-1β and TNF-α; consistently, C-reactive protein and leukocyte count decreased in resveratrol-treated patients. This study is relevant as it demonstrates, using a robust methodology, that resveratrol may have anti-inflammatory effects in a clinical model of serious viral infection.

In this regard, a recent review outlined the antiviral activity of resveratrol against several viruses, including double-stranded linear or partially double-stranded circular DNA viruses, negative-sense single-stranded RNA viruses with non-segmented or segmented genomes, and positive-sense single-stranded RNA viruses [44]. Another review reinforced the concept that resveratrol has relevant antiviral activity and also modulates the gut microbiota [45]. In particular, resveratrol may reshape the gut microbiota by reducing opportunistic taxa and enriching beneficial bacteria, such as *Bifidobacterium* and *Lactobacillus*. This microbiota change promotes short-chain fatty acid production, increases the barrier integrity, and, finally, contributes to dampening inflammatory events linked to viral infections. These positive effects depend on a specific mechanism of action of resveratrol, which deacetylates sirtuins [46]. Sirtuins are a family of essential proteins that regulate vital processes such as energy metabolism, DNA repair, oxidative stress defence, and inflammation, acting as “regulators of cellular survival” [47]. Sirtuins are present in all organisms, and there are seven types in humans (SIRT1-7), each with specific functions, positively influencing lifespan and preventing chronic diseases [48]. Cellular stressors, such as infections, inflammation, and oxidative stress, trigger sirtuin production. However, polyphenols, including resveratrol, are powerful inducers of sirtuins. In this regard, a recent study showed that resveratrol may increase SIRT1 expression, a central NAD+-dependent deacetylase, in a viral infection model [49]. SIRT1 may be viewed as a rheostat of innate immunity homeostasis; thus, resveratrol may have potential benefits in viral infections. This set of clinical and experimental evidence confirms findings from a study exploring the antiviral effects of resveratrol in combination with CMBG [19]. H1HeLa cell cultures and ex vivo nasal epithelial cells infected by HRV-16 were tested.

Resveratrol significantly inhibited rhinovirus replication and virus-mediated expression of pro-inflammatory cytokines IL-6 and IL-8, and the chemokine RANTES. In addition, resveratrol reversed the expression of ICAM-1, a molecule deeply implicated in allergic inflammation and a primary receptor for rhinoviruses. Allergen exposure triggers an inflammatory cascade that involves the recruitment of inflammatory cells [50]. Adhesion machinery regulates cellular trafficking from endothelia to the respiratory mucosa. In this regard, ICAM-1 plays a pivotal role by facilitating mucosal infiltration by leukocytes, mainly eosinophils, as it is the ligand for LFA-1, an integrin expressed on leukocytes [51]. Consistently, ICAM-1 expression on epithelial cells is closely associated with allergen exposure as it disappears when the allergic subjects are not exposed to the causal allergen [52].

As a consequence, inhibiting ICAM-1 expression reduces the likelihood of eosinophil infiltration, as documented for antiallergic molecules [53]. In other words, if the nasal epithelium expresses fewer ICAM-1 adhesion molecules, there will be less chance of it being infiltrated by inflammatory cells that express its ligand, i.e., integrins. Similarly, as ICAM-1 is the primary receptor for rhinoviruses (the main pathogens of the common cold), lower expression of the viral receptor will make the subject less susceptible to infection [54]. Therefore, these studies may hypothesize that intranasal resveratrol can, on the one hand, reduce allergic inflammation and, on the other, prevent viral rhinitis.

As regards the antifungal activity of resveratrol, plants naturally produce resveratrol as a physiological phytoalexin to defend against fungal damage [55]. There is substantial evidence that resveratrol can inhibit several fungi, including dermatophytes such as *Trichophyton mentagrophytes*, *Trichophyton tonsurans*, *Trichophyton rubrum*, *Epidermophyton floccosum*, and *Microsporum gypseum* [56]. In addition, resveratrol, when adequately delivered, may also inhibit *Candida albicans* [57,58].

The antagonistic activity of resveratrol towards protozoa is well documented, with evidence that it may hinder various microorganisms [59]. In fact, resveratrol may inhibit distinct *Leishmania* species, *Schistosoma mansoni*, *Toxoplasma gondii*, *Trichomonas vaginalis*, *Trypanosoma cruzi*, and various *Amoeba* species [60,61,62,63,64,65].

Therefore, considering this body of evidence, resveratrol, due to its intrinsic phytoalexin properties, can combat numerous pathogens from various classes of microorganisms, namely viruses, bacteria, fungi, and protozoa.

### 4.2. Anti-Inflammatory Activity of Resveratrol

There is a wealth of experimental and clinical data on this anti-inflammatory activity, gathered from in vitro, animal, and human studies, which is truly remarkable and has been reported in numerous recent reviews [66,67,68,69]. As these reviews are very recent and exhaustive, we synthesize the main aspects of their anti-inflammatory activity, referring to them for details.

Resveratrol has powerful anti-inflammatory properties, acting by inhibiting signalling pathways such as NF-κB and reducing the release of pro-inflammatory molecules, counteracting acute and chronic inflammatory events, and activating sirtuins, involved in cellular repair, and the AMPK protein, crucial for cellular energy balance.

The anti-inflammatory mechanism of action includes different pathways. Resveratrol inhibits cytokine production, as clinical and in vitro studies have highlighted its ability to modulate the inflammatory response by reducing levels of circulating pro-inflammatory mediators, particularly IL-6 and TNF-α, and, in turn, increasing the anti-inflammatory cytokine IL-10. This modulation is particularly relevant in conditions associated with chronic inflammation.

Resveratrol modulates the immune response, suggesting its potential use in diseases characterized by excessive inflammatory reactions, such as certain complications related to viral infections (e.g., hyperinflammation during the cytokine storm).

Resveratrol protects tissue integrity by inhibiting inflammatory, damaging, and remodelling processes. Resveratrol also works by inhibiting inflammatory signals: it blocks the NF-κB pathway, a key regulator of the inflammatory response, thereby reducing the production of pro-inflammatory mediators. In addition, resveratrol inhibits macrophage and lymphocyte activation and can halt abnormal cell growth. To summarize, a recent in vitro and mouse study investigated the anti-inflammatory and antiviral effects of an innovative resveratrol formulation [23]. In this regard, emerging interest has focused on small-molecule self-assembled nanotechnology to achieve effective delivery, increased drug loading capacity, improved solubility, and stability [70]. The investigators prepared sonicated-assisted, self-assembled resveratrol nanoparticles and used them for nebulization in a mouse model of RSV (Respiratory Syncytial Virus)-induced pneumonia and in in vitro studies. The in vivo results demonstrated that this resveratrol formulation reduced viral load, thereby inhibiting viral replication. The in vitro findings showed that resveratrol inhibited NO release (antioxidant activity) and reduced the expression of pro-inflammatory cytokines IL-6 and TNF-α. These results confirmed that resveratrol may attenuate lung inflammation in the animal model of viral pneumonia.

### 4.3. Antioxidant Activity of Resveratrol

Resveratrol is a powerful antioxidant that fights free radicals, reducing oxidative stress and protecting cells from ageing and environmental damage, improving respiratory, skin, cardiovascular, and metabolic health [71,72,73,74,75,76,77]. Actually, antioxidant activity is the primary property of resveratrol, as it is produced to counteract plant oxidative stress. The literature on the antioxidant effects of resveratrol is vast, with over 13,000 scientific articles on PubMed when searching for this keyword. Again, we refer you to the most recent reviews on this topic for analytical details.

Resveratrol exerts antioxidant activity through multiple mechanisms, including activating antioxidant enzymes (Nrf-2) and inhibiting inflammatory pathways (NF-kB). In particular, resveratrol neutralizes free radicals, directly capturing them. Resveratrol also stimulates the endogenous production of antioxidant enzymes (such as those regulated by Nrf-2) and activates protective proteins (SIRT1). As a result, the antioxidant properties of resveratrol may be beneficial for all body systems and metabolism, as well as for several diseases.

In the current context of respiratory infections and allergic diseases, resveratrol’s antioxidant activity could be clinically relevant, as it might interrupt the vicious cycle of the inflammatory cascade. However, adequate studies should confirm this possibility.

### 4.4. Immunomodulant Activity of Resveratrol

Resveratrol has a marked immunomodulatory activity, acting as a powerful antioxidant and anti-inflammatory that modulates immune responses, reduces pro-inflammatory cytokines (such as IL-6 and TNF-α), inhibits the activation of immune cells such as macrophages and lymphocytes, and supports the function of defence cells (NK, macrophages, and neutrophils), counteracting chronic inflammation and viral infections and helping to regulate autoimmune diseases and allergies by influencing key cellular pathways such as NF-κB and activating sirtuins for longevity [78,79,80,81,82,83].

The mechanisms of immunomodulatory action include combined multifaceted effects, such as (i) anti-inflammatory action (modulating cell signalling pathways (NF-κB and MAPK)) to reduce the production of pro-inflammatory cytokines (IL-6 and TNF-α) and C-reactive protein; (ii) powerful antioxidant effects (combating oxidative stress, protecting cells and supporting mitochondrial function); and (iii) immune cell activation (recruiting and activating macrophages, lymphocytes and natural killer cells, and boosting defence against pathogens) and sirtuin regulation (activating enzymes involved in DNA repair, longevity and cellular homeostasis).

In summary, resveratrol does not simply act as a stimulant or suppressor, but as a modulator that may help balance the body’s immune response, making it an active ingredient of potential interest for supporting immune health. However, many of its biological activities are still the subject of scientific research for full validation.

### 4.5. Antiallergic Activity of Resveratrol

Resveratrol has significant antiallergic activity because it acts as a potent anti-inflammatory and antioxidant, modulating the immune response: it reduces the production of inflammatory mediators (cytokines) and interferes with allergic processes, alleviating symptoms such as itching, sneezing, and nasal congestion, especially in specific formulations such as nasal sprays for allergic rhinitis [84,85,86,87,88,89,90,91].

Resveratrol exerts antiallergic activity by reducing pro-inflammatory cytokines (such as IL-4, IL-6, and TNF-α) and C-reactive proteins, and blocking cell signalling pathways (Nrf2, NF-κB, and MAPK) and mast cell activation, phenomena involved in allergic reactions. In addition, resveratrol counteracts oxidative stress, a trigger and aggravating factor of allergies, and regulates the activity of immune cells, such as macrophages and lymphocytes, boosting the defence system and reducing allergic hyper-reactivity. In fact, resveratrol-based nasal spray formulations (often combined with CMBG) have shown significant benefits in reducing nasal symptoms and related infections, even in children. In this regard, a recent systematic review and meta-analysis concluded that polyphenolic compounds, particularly resveratrol, may be a complementary option for managing patients with allergic rhinitis [92].

### 4.6. Direct Molecular Targets of Resveratrol

Resveratrol exerts all these effects by targeting key proteins that regulate inflammation, primarily by activating anti-inflammatory pathways SIRT1 (Silent Information Regulator 1) and AMPK (AMP-activated protein kinase) and inhibiting pro-inflammatory ones (NF-κB, COX-2, and NLRP3) [93,94,95]. Resveratrol directly activates SIRT1, which subsequently deacetylates and inhibits pro-inflammatory proteins. Resveratrol also inhibits the activation of NF-κB, a major transcription factor for pro-inflammatory cytokines, by preventing the degradation of IκBα and reducing p-NF-κB, exerting protective effects against inflammatory damages [96]. Resveratrol activates AMPK, which acts as an energy sensor that can reduce inflammation, e.g., by activating autophagy and protecting from physical damage [97]. Resveratrol suppresses the expression of COX-2, an enzyme responsible for inflammation and pain, as it regulates the production of arachidonic acid metabolites, i.e., prostaglandins, leukotrienes, and thromboxane [98]. Resveratrol inhibits the assembly and activation of the NLRP3 inflammasome, a multiprotein complex that induces inflammatory responses; thus, resveratrol may dampen NLRP3-driven inflammatory conditions, such as gout [99]. Resveratrol targets and inhibits the phosphorylation of AKT1, which is involved in initiating inflammation; so, it may mitigate the cascade of inflammatory events [100]. Resveratrol also impacts enzymes and factors like iNOS (inducible nitric oxide synthase), TNF-α (tumour necrosis factor-alpha), IL-6, IL-1β, p300, and NADPH oxidase [101,102,103]. As a result, resveratrol may act on different inflammatory targets, providing multifaceted activity.

Therefore, by modulating the key proteins involved in the inflammatory process, resveratrol can effectively lower the production of pro-inflammatory mediators and cytokines, offering a potential therapeutic approach to managing inflammatory conditions associated with infection, allergy, and oxidative stress. However, documented evidence is necessary to confirm this hypothesis.

## 5. Intranasal CMBG–Resveratrol: In Vitro and Preclinical Evidence

Intranasal CMBG–resveratrol has been tested in various studies, as summarized in Table 1. A study explored its capability of inhibiting inflammatory events consequent experimental vital infection [19]. The same study demonstrated that CMBG–resveratrol reduced ICAM-1 expression: an adhesion molecule involved in allergic inflammation and rhinovirus infection. Laboratory studies investigated its stability, solubility, and physical characteristics to be administrated in aerosol formulation [13,18,20]. An animal and in vitro study showed that CMBG–resveratrol reduced viral load and inflammatory biomarkers [23].

These data have a limited evidence level as they were conducted in in vitro and animal models. As a consequence, the results should be considered preliminary to clinical investigations.

## 6. Intranasal CMBG–Resveratrol: Clinical Evidence

Resveratrol, thanks to its multiple activities (antioxidant, anti-inflammatory, antimicrobial (overall antiviral), immunomodulatory, and antiallergic), has been investigated in several clinical studies. In particular, it has been evaluated primarily in its intranasal formulation, combined with CMBG, to optimize stability and duration of action, with the main areas of study being respiratory infections, allergies, and nasal surgery. In this regard, three recent papers have analyzed in detail the most relevant published studies [104,105,106]. However, these articles did not consider all globally published studies on the intranasal combination of CMBG–resveratrol, evaluate the peculiar activities provided by the components of the intranasal combination, and underline their clinical relevance.

Table 2 summarizes these clinical studies. Presently, we synthesize the leading outcomes of these clinical studies, subdivided by therapeutic area.

### 6.1. Respiratory Infections

Regarding respiratory infections, four clinical trials were conducted in children [107,108,109,110].

The first randomized controlled study included 82 children suffering from acute rhinopharyngitis and recurrent respiratory infections (RRIs) [107]. The children were stratified into two subgroups: one treated with CMBG–resveratrol (one spray per nostril/twice daily for 20 days) and the other with isotonic saline. The standard therapy for all patients was nebulization with thiamphenicol-acetyl cysteine-beclomethasone for ten days. Treatment with CMBG–resveratrol significantly reduced the severity of respiratory symptoms and rescue medication use.

The second randomized, placebo-controlled study included 76 children with persistent allergic rhinitis and a history of frequent respiratory infections [108]. Children were subdivided into an active group and a placebo group. Actively treated children received CMBG–resveratrol (two sprays per nostril/three times daily for 2 months). The active treatment significantly reduced the intensity of nasal and bronchial (wheezing) symptoms, the duration of fever, cough frequency, the use of bronchodilators, and school absence.

The third placebo-controlled double-dummy study evaluated 89 infants (0–6 months of age) with acute respiratory infection (mainly the common cold) who were treated with resveratrol–CMBG drops (three drops per nostril, four times daily for 1 week) or placebo [109]. Active treatment significantly reduced the severity of nasal symptoms.

The last placebo-controlled study included 39 children with an RRI; the active group received resveratrol four times daily for 1 week during each respiratory infection [110]. Resveratrol–CMBG significantly reduced wheezing episodes, hospitalizations, and oral corticosteroid use.

A very recent randomized placebo-controlled study was conducted in four Italian centres enrolling children aged 2–6 years with recurrent respiratory infections (unpublished data). The results preliminarily confirmed that a once-daily 12-week course with resveratrol–CMBG nasal spray provided fewer symptomatic days, fewer infectious episodes, and reduced antibiotic use compared with placebo, corresponding to an approximate 25–30% reduction in symptom burden.

### 6.2. Allergic Rhinitis

Three clinical studies investigated patients with allergic rhinitis [108,111,112].

The first study presented above demonstrated that CMBG–resveratrol significantly diminished the allergic symptoms [108].

The second placebo-controlled study enrolled 68 children with seasonal allergic rhinitis [111]. Children treated with CMBG–resveratrol received two nasal sprays three times daily for 2 months, including during the pollen season. The treatment significantly reduced symptom severity and antihistamine use on demand.

The third placebo-controlled, double-blinded study considered 151 adult patients with persistent allergic rhinitis [112]. The study consisted of three arms: placebo, intranasal budesonide, and intranasal resveratrol (extracted from *Polygonum cuspidatum*) diluted in an isotonic solution to 0.1%. Patients took two sprays into one nostril three times daily for 1 month. Resveratrol improved patients’ allergic symptoms and quality of life and significantly reduced IgE, IL-4, TNF-α, and eosinophil levels in peripheral blood. Thus, this preliminary study provided positive clinical outcomes consistent with inflammation dampening.

### 6.3. Nasal Surgery

One controlled study included 70 patients with chronic nasal obstruction who were candidates for functional endoscopic sinus surgery [113]. CMBG–resveratrol treatment was used as follows: one puff for the nostril three times per day for one month from the second post-surgery week. The control group took only saline solution nasal irrigation. The active treatment significantly improved the SNOT-20 (sino-nasal outcome test) score and endoscopic findings, including mucosal edema and secretions, as assessed by the ENS (endoscopic nasal score).

### 6.4. Safety Aspects

All clinical studies reported an optimal safety profile of the intranasal combination CMBG-resveratrol as no clinically relevant adverse events were reported. In addition, long term follow-up data confirmed the safety of this combination.

### 6.5. Limitations of Current Evidence and Unresolved Issues

Actually, these studies show relevant heterogeneity, particularly with respect to differences in the disease models, patient age groups (infants versus adults), dosing regimens, schedules, clinical endpoints, and placebo or comparator interventions. In addition, these studies had small sample sizes, homogeneous populations, and short follow-ups. Consequently, such variability and quality of evidence may limit the overall generalizability and strength of the clinical conclusions.

Consequently, a series of unresolved issues deserve adequate attention. In this regard, new clinical studies should be performed considering robust methodology, including sample size calculation, randomization, double-blind design, homogeneous populations and schedules.

## 7. Conclusions

Resveratrol is a polyphenol characterized by significant antioxidant, antimicrobial (mainly antiviral), anti-inflammatory, immunomodulatory, and antiallergic properties. For these reasons, it has been used to manage many diseases, including respiratory and allergic infections. However, resveratrol has low oral bioavailability. For these reasons, an innovative formulation has been designed and developed that complexes trans-resveratrol with carboxymethyl beta-glucan to ensure adequate stability and availability in the airways, as it is available as a nasal spray.

This combination was therefore the subject of in-depth preliminary in vitro studies that first assessed its stability and solubility and then its antiviral and anti-inflammatory activity. A subsequent series of randomized, controlled clinical trials confirmed the efficacy and safety of the resveratrol–CMBG combination in various clinical conditions, including respiratory infections, allergic rhinitis, and nasal surgery. However, this narrative review reports a series of results whose interpretation requires a certain degree of caution, considering the limitations of these various studies. On the other hand, taken together, these findings may suggest that intranasal resveratrol–CMBG could be a promising option for the management of upper airway diseases.

## Figures and Tables

**Table 1:** In vitro and preclinical evidence. Preclinical studies established that CMBG–resveratrol maintains biological activity while improving delivery characteristics. The combination demonstrates effects across multiple therapeutic mechanisms.

Ref.	Study Model	Key Findings	Clinical Relevance
[19]	H1HeLa cells + HRV-16	Inhibited rhinovirus replication, reduced IL-6, IL-8, RANTES expression	Antiviral mechanism against common cold pathogens
[19]	Ex vivo nasal epithelial cells	Reduces ICAM-1 expression, blocked viral receptor	Prevents viral entry and reduces allergic inflammation
[13,18]	Stability studies	CMBG maintained resveratrol’s stability and solubility in aqueous solutions	Enables effective topical intranasal delivery
[20]	Aerosol formulation	Mass median aerodynamic diameter suitable for entire respiratory tree	Potential for lower airway delivery
[23]	Mouse RSV pneumonia model	Reduced viral load, inhibited NO release, decreased IL-6/TNF-a	Demonstrates in vivo anti-inflammatory and antiviral effects
[18]	Photochemical stability	Enhanced bioavailability and long-term stability with CMBG	Ensures consistent therapeutic efficacy

**Table 2:** Clinical Studies: proven efficacy and safety. Seven controlled clinical trials demonstrate consistent efficacy and safety of intranasal resveratrol–CMBG across respiratory infections, allergic rhinitis, and post-surgical recovery in both pediatric and adult populations.

Ref	Study	Population	Treatment Protocol	Key Outcomes
[107]	RCT (*n* = 82)	Children with acute rhinopharyngitis and RRI	1 spray/nostril, 2x daily, 20 days	Significantly reduced respiratory symptom severity and rescue medication use vs. saline
[108]	RCT (*n* = 76)	Children with persistent allergic rhinitis + frequent infections	2 sprays/nostril, 3x daily, 2 months	Reduced nasal/bronchial symptoms, fever duration, cough frequency, bronchodilator use, and school absence
[109]	Double-blind (*n* = 89)	Infants 0–6 months with acute respiratory infection	3 drops/nostril, 4x daily, 1 week	Reduced wheezing episodes, hospitalizations, and oral corticosteroid use
[110]	Placebo-controlled (*n* = 39)	Children with RRI	4x daily, 1 week per infection episode	Reduced wheezing episodes, hospitalizations, and oral corticosteroid use
[111]	Placebo-controlled (*n* = 68)	Children with seasonal allergic rhinitis	2 sprays, 3x daily, 2 months (pollen season)	Reduced symptom severity and on-demand antihistamine use
[112]	Double-blind (*n* = 151)	Adults with persistent allergic rhinitis	2 sprays/nostril, 3x daily, 1 month (0.1% resveratrol)	Improved symptoms and QoL; reduced IgE, IL-4, TNF-α, and eosinophils vs. placebo and comparable to budesonide
[113]	Controlled (*n* = 70)	Adults with post-functional endoscopic sinus surgery	1 puff/nostril, 3x daily, 1 month (starting week 2 post-op)	Improved SNOT-20 scores and endoscopic findings (mucosal edema, secretions) vs. saline irrigation

RCT = randomized clinical trial; RRI = recurrent respiratory infection; QoL = quality of life.

## Data Availability

No new data were created or analyzed in this study.
